# Diverticulum of the Œsophagus

**Published:** 1891-05

**Authors:** A. W. Clement


### DIVERTICULUM OF THE ŒSOPHAGUS.

By A. W. Clement, V. S.

Clinical History.—The animal has been in the possession of its present owner for about one year. He had been driven by the same coachman all of this time. During the first six months he was kept at livery. For the past six months the coachman has taken care of him. While the animal was at livery the groom said that he had a *strangling cough*. Two days after he came under the coachman's care he noticed, on entering the stable in the morning, that the animal was apparently in distress. On examination he found that the animal had left most of his supper in the manger. That when he attempted to swallow water it returned by the nose and mouth. He then noticed a *lump* in the horse's throat. The attack lasted for about twenty-four hours, during which time the animal suffered great agony. He was very restless and breathed with difficulty. At the end of twenty-four hours the *lump* disappeared and the animal was apparently as well as ever. He had three subsequent attacks during the last of which he died.

AUTOPSY.—Subject, a thoroughbred chestnut gelding, five years old, of about 1000 lbs. weight, and in good condition. Autopsy nine hours after death. *General inspection.* Nothing abnormal to be noticed. No distension of the abdomen. *Special inspection.* Mucous membrane of nose presents striated and punctiform hemorrhages covering an area 5 cm. long and 3 cm. wide in widest portion. This condition in both nostrils. Mucous membrane of mouth pale. There are two superficial, greenish-looking ulcers on border of inferior maxilla, one on either side. Also two similar ulcers in corresponding positions on superior maxilla. "These ulcers were caused by the mouth speculum used shortly before death." Conjunctival mucous membranes somewhat yellow in appearance. There is no elevation to be seen over region of the œsophagus.

SUBCUTANEOUS.—Muscle dry, somewhat yellowish in color. Lymphatic glands reddened, especially in axilla.

ABDOMEN.—No fluid. Peritoneum normal, organs normal.

THORAX.—No fluid, no adhesions. Lung feels solid in lower portion of the posterior lobe, on right side. A mass about as large as a goose's egg can be seen in the medio-stinum, just at the base of the heart. Further dissection shows this mass to be in the course of the œsophagus. Heart and lungs with nearly all of the trachea and œsophagus removed *en masse*. *Pericardium* contains no fluid in cavity. Surface smooth and glistening. *Epicardium* smooth and glistening. There are a considerable number of sub-epicordial striated hemorrhages. Heart muscle firm and of normal color. Cavities filled with dark colored coagulated blood. The right heart contains rather more blood than does the left heart. *Endocardium* normal, valves normal. No sub-endocardial hemorrhages. Aorta normal. *Lungs* and *Pleura*. Right lung crepitant throughout. Pleural surface smooth and glistening. Left lung, at lower border of the posterior lobe in an area 15 cm. long by 7 cm. wide; in the widest portion is solid. The plural surface over entire lung is smooth and glistening. The solid area on section presents a grayish-red appearance. The bronchi contains small particles of food; the mucous membrane is of normal appearance but there are many striated hemorrhages beneath the mucous membrane. The trachea contains some undigestible particles of food, is greenish in color on the mucous surface and there are some circumscribed ulcerations of the mucous. *Oesophagus.* There is dilatation of the tube to two or three times its normal size. The dilatation measures 60 cm. in length and from 10 to 13 cm. in width. It begins and ends quite abruptly. It starts from about opposite the third cervical vertebræ. 45 cm., from the beginning of the dilatation is a diverticulum projecting from the outer and slightly toward the lower border of the tube. This diverticulum measures 5 cm. in length, and 2.5 cm. in depth. It is empty, soft and very much thinner than the wall of the tube ajacent.

On section of the œsophagus there is seen to be a loss of muscle fibre with protrusion and dilatation of the mucous membrane to form the sack or diverticulum. The mucous membrane throughout the dilated portion of the tube is greenish in color. The muscle substance is flaby. The mass in the thoracic portion of the œsophagus is found to be a boltis of masticated food. THROAT and MOUTH organs normal. BRAIN and SPINAL CHORD, not examined.

